# Wild-Type and Non-Wild-Type Mycobacterium tuberculosis MIC Distributions for the Novel Fluoroquinolone Antofloxacin Compared with Those for Ofloxacin, Levofloxacin, and Moxifloxacin

**DOI:** 10.1128/AAC.00393-16

**Published:** 2016-08-22

**Authors:** Xia Yu, Guirong Wang, Suting Chen, Guomei Wei, Yuanyuan Shang, Lingling Dong, Thomas Schön, Danesh Moradigaravand, Julian Parkhill, Sharon J. Peacock, Claudio U. Köser, Hairong Huang

**Affiliations:** a National Clinical Laboratory on Tuberculosis, Beijing Key Laboratory on Drug-Resistant Tuberculosis Research, Beijing Chest Hospital, Capital Medical University, Beijing Tuberculosis and Thoracic Tumor Institute, Beijing, China; b Department of Medical Microbiology, Linköping University Hospital, Linköping, Sweden; c Department of Clinical Microbiology and Infectious Diseases, Kalmar County Hospital, Kalmar, Sweden; d Wellcome Trust Sanger Institute, Hinxton, United Kingdom; e Department of Medicine, University of Cambridge, Cambridge, United Kingdom; f London School of Hygiene & Tropical Medicine, London, United Kingdom

## Abstract

Antofloxacin (AFX) is a novel fluoroquinolone that has been approved in China for the treatment of infections caused by a variety of bacterial species. We investigated whether it could be repurposed for the treatment of tuberculosis by studying its *in vitro* activity. We determined the wild-type and non-wild-type MIC ranges for AFX as well as ofloxacin (OFX), levofloxacin (LFX), and moxifloxacin (MFX), using the microplate alamarBlue assay, of 126 clinical Mycobacterium tuberculosis strains from Beijing, China, of which 48 were OFX resistant on the basis of drug susceptibility testing on Löwenstein-Jensen medium. The MIC distributions were correlated with mutations in the quinolone resistance-determining regions of *gyrA* (*Rv0006*) and *gyrB* (*Rv0005*). Pharmacokinetic/pharmacodynamic (PK/PD) data for AFX were retrieved from the literature. AFX showed lower MIC levels than OFX but higher MIC levels than LFX and MFX on the basis of the tentative epidemiological cutoff values (ECOFFs) determined in this study. All strains with non-wild-type MICs for AFX harbored known resistance mutations that also resulted in non-wild-type MICs for LFX and MFX. Moreover, our data suggested that the current critical concentration of OFX for Löwenstein-Jensen medium that was recently revised by the World Health Organization might be too high, resulting in the misclassification of phenotypically non-wild-type strains with known resistance mutations as wild type. On the basis of our exploratory PK/PD calculations, the current dose of AFX is unlikely to be optimal for the treatment of tuberculosis, but higher doses could be effective.

## INTRODUCTION

In 2009, the Chinese State Food and Drug Administration granted marketing approval for the new fluoroquinolone antofloxacin hydrochloride (here referred to as antofloxacin [AFX]), a derivative of levofloxacin (LFX) (1, 2). Its intended uses are for the treatment of (i) acute bacterial exacerbations of chronic bronchitis due to Klebsiella pneumoniae, (ii) acute pyelonephritis and cystitis due to Escherichia coli, and (iii) wound infection and multiple epifolliculitis due to Staphylococcus aureus or coagulase-negative staphylococci (1). However, given that AFX has activity against a wider array of bacterial pathogens and other fluoroquinolones are used for treatment of tuberculosis, we wanted to investigate its *in vitro* activity against Mycobacterium tuberculosis strains from China (1). Moreover, we studied the degree of cross-resistance to fluoroquinolones that are already being used to treat tuberculosis (i.e., ofloxacin [OFX], LFX, and moxifloxacin [MFX]) on a phenotypic as well as a genotypic level to assess whether current genotypic drug susceptibility testing (DST) assays could be used to detect resistance to AFX and whether AFX might be an option for the treatment of infections caused by strains that are resistant to these existing fluoroquinolones.

## MATERIALS AND METHODS

### Study setting and bacterial strains.

We studied 126 M. tuberculosis complex strains that were collected from the National Clinical Laboratory on Tuberculosis, Beijing Chest Hospital, between January and March 2014 from retreatment patients with presumed multidrug-resistant (MDR) tuberculosis (i.e., resistance to rifampin and isoniazid), which included 45 pansusceptible M. tuberculosis strains, 49 MDR M. tuberculosis strains, and 17 extensively drug-resistant M. tuberculosis strains (i.e., MDR M. tuberculosis strains with additional resistance to OFX and amikacin or capreomycin), as well as 3 strains that were monoresistant to OFX (Sigma-Aldrich, St. Louis, MO, USA), as determined using the absolute concentration method on Löwenstein-Jensen medium (LJ) with 2 μg/ml as the critical concentration (CC). The M. tuberculosis laboratory strain H37Rv (ATCC 27294) served as a negative control.

### MIC testing.

We determined the MICs for OFX, LFX (Sigma-Aldrich, St. Louis, MO, USA), MFX (Bayer Pharmaceutical Corporation, Leverkusen, Germany), and AFX (Anhui Huanqiu Pharmaceutical Co., Hefei, China) using the microplate alamarBlue assay (MABA) in 2-fold dilutions ranging from 16 to 0.032 μg/ml (3, 4). Drug powder was dissolved in 1% NaOH at a concentration of 10 mg/ml, and different aliquots were prepared and stored at −70°C. All the working solutions were freshly prepared before use. All the strains were subcultured onto LJ slopes for 3 weeks. Bacterial suspensions were prepared using 5% (vol/vol) Tween 80 in 0.9% NaCl, and the turbidity was adjusted to a 1 McFarland turbidity standard. Suspensions were further diluted (1:25) with 7H9 broth. H37Rv was used as a control.

### Genotypic analyses.

We sequenced the quinolone resistance-determining regions (QRDRs) of *gyrA* (*Rv0006*) and *gyrB* (*Rv0005*) and called mutations relative to the sequence of the H37Rv reference genome (GenBank accession number AL123456.3) using the 2002 numbering for *gyrB* (5 – 7). We usually sequenced isolates recovered from the drug-free LJ slopes, but where no resistance mutations were found in phenotypically resistant strains, sequencing was repeated with isolates recovered from the OFX-containing LJ slope to detect low-frequency mutations (8, 9). Strains belonging to the East Asian lineage were identified on the basis of RD105 (10).

## RESULTS

**FIG 1:**
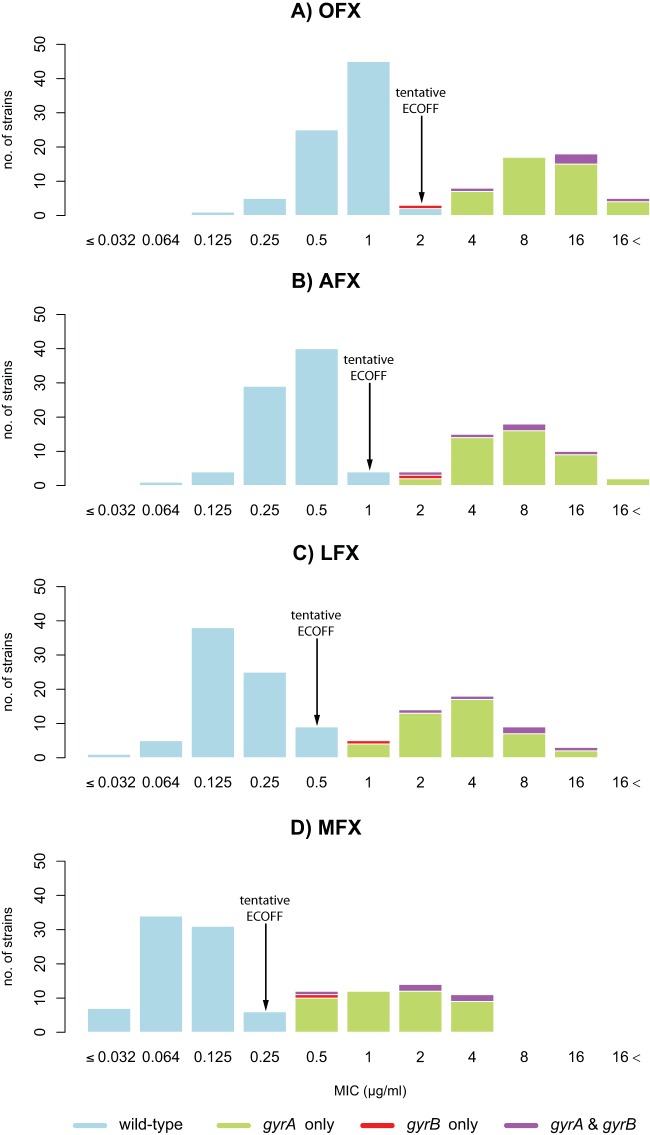
Wild-type and non-wild-type MIC distributions for the four fluoroquinolones under investigation relative to their *gyrA* and *gyrB* genotypes (see Table S1 in the supplemental material). The tentative ECOFF represents the upper limit of the wild-type distribution. All clinical strains, with the exception of H37Rv, harbored the *gyrA* S95T mutation that is known not to confer fluoroquinolone resistance and was consequently excluded from the analysis (13).

A total of 92.9% (117/126) of the strains in this study belonged to the East Asian lineage (see Table S1 in the supplemental material) (11). We found that the MIC distributions for all four fluoroquinolones were bimodal (Fig. 1A to D), where the more susceptible of the two distributions represented the phenotypically wild-type distributions, whereas the remaining strains were, by definition, phenotypically non-wild type. Based on visual inspection, we therefore set tentative epidemiological cutoff values (ECOFFs) for MIC determination using the MABA method at 2, 1, 0.5, and 0.25 μg/ml for OFX, AFX, LFX, and MFX, respectively (12). Not all phenotypically wild-type strains were identical genotypically (i.e., all 126 Chinese strains harbored the known *gyrA* S95T mutation that does not correlate with resistance [7, 13]), but after the exclusion of this polymorphism, we found a nearly perfect correlation between the tentative ECOFFs and nonsynonymous mutations in the two subunits of DNA gyrase, encoded by *gyrA* and *gyrB*.

All *gyrA* mutations detected in this study were classical resistance mutations that fell into the QRDR and resulted in an MIC increase above the tentative ECOFF for all four fluoroquinolones (Fig. 1; see also Table S1 in the supplemental material) (7, 14). This was in line with the fact that all *gyrA* mutants tested resistant to OFX on LJ, although retesting of seven strains that were initially discrepant was required to achieve complete agreement (Table 1). In line with a recent systematic review, the D94G and A90V mutations were the most frequent and the second most frequent mutations, respectively, whereas other changes (e.g., G88C) occurred in only a single strain (15). Theoretically, all of these mutations could have been detected with the genotypic DST assays of Hain Lifescience, Nipro, and YD Diagnostics, whereas the assays of AID and Seegene would have missed the two resistant strains with mutations at codon 88 (see Table S1 in the supplemental material) (16 – 22). In practice, however, some resistance mutations might have been missed, given that the detection limits of these assays, albeit unknown, are almost certainly higher than the critical proportion of 1% (e.g., strain 14140 was heteroresistant, and its D94G mutation was detectable only using Sanger sequencing of an isolate from the drug-containing slope [see Table S1 in the supplemental material]) (23 – 25).

**TABLE 1:** Initial and repeat LJ DST and MABA MIC results for the seven strains for which there was disagreement between the different methods during the initial round of testing^*a*^

Strain	OFX LJ DST result	MABA MIC (μg/ml)				Genotype^*b*^	
		OFX	AFX	LFX	MFX	*gyrA*	*gyrB*
14170	**R**	2	0.25	0.125	0.125	WT	WT
	S	0.5	0.5	0.25	0.25		
12657	**R**	2	1	0.5	0.25	WT	WT
	S	1	1	0.5	0.25		
14130	**R**	2	0.5	0.25	0.125	WT	WT
	S	1	1	0.5	0.25		
14132	**R**	0.5	0.5	0.125	0.125	WT	WT
	S	1	0.5	0.5	0.25		
14150	**R**	2	**2**	1	0.5	WT	WT
	S	1	1	0.5	0.25		
14175	**R**	2	0.5	0.25	0.125	WT	WT
	S	0.5	0.5	0.25	0.125		
14198	R	**2**	4	2	1	D94A	WT
	R	8	8	4	2		
14117	S	2	1	0.5	0.25	WT	T500N
	S	2	**2**	**1**	**0.5**		

a In each case, the repeat results are shown in Fig. 1 and listed in Table S1 in the supplemental material. MICs above the ECOFF (i.e., phenotypically non-wild-type results) are underlined. All of these discrepancies, which are shown in bold, were resolved upon retesting. In contrast, strain 14117 was retested because the initial MICs and the previous literature suggested that the MICs were close to the ECOFFs, which retesting supported.

b Excluding the *gyrA* S95T polymorphism. WT, wild type.

As expected, *gyrB* mutations were rare and usually coincided with *gyrA* mutations (in 5/6 cases); thus, they did not improve markedly the sensitivity of detecting phenotypically non-wild-type strains (48/49 strains had a *gyrA* mutation) (15). Strain 14117 was the sole exception. It harbored only a *gyrB* mutation (T500N), was found to be susceptible to OFX on LJ, and had MABA MICs that corresponded to the aforementioned ECOFFs for the four respective fluoroquinolones (Table 1). The mutation in question fell just outside of the *gyrB* QRDR, as defined by Maruri et al. (7), which spans codons 461 to 499, but inside the QRDR based on the findings of Pantel et al. (26), which extends to codon 501. Using the recently developed version 2 of the Hain Lifescience Genotype MTBDR*sl* assay, which covers codons 497 to 502 of *gyrB*, an isolate with this mutation would also have been interpreted to be resistant (22). We therefore repeated DST for this strain, whereupon the MICs for AFX, LFX, and MFX increased by 1 doubling dilution and the strain consequently became phenotypically non-wild-type, whereas the OFX MIC and LJ result remained unchanged (Table 1).

## DISCUSSION

The aim of DST is usually to distinguish resistant strains, patients infected with which are likely to fail treatment, from susceptible strains, patients infected with which have a high likelihood of clinical success (an intermediate category is sometimes possible) (27). The clinical breakpoints (known as critical concentrations [CCs] in the tuberculosis field) employed for this purpose should be based on clinical, pharmacokinetic/pharmacodynamic, and, ideally, clinical outcome data, which, for a variety of reasons, are difficult to obtain for tuberculosis drugs (27). As a result, an important aim of DST for the majority of tuberculosis drugs is to distinguish wild-type from non-wild-type strains [i.e., strains with elevated MICs compared with those for strains that (i) have never been exposed to the agent or class of agent in question and (ii) are not intrinsically resistant] using the ECOFF, which represents the highest concentration of the wild-type distribution determined by modern microbiological principles pioneered by the European Committee on Antimicrobial Susceptibility Testing (EUCAST) (12, 23, 27 – 30). In other words, the ECOFF represents the lowest possible CC and some non-wild-type strains might remain treatable, as proposed for MFX, albeit on the basis of limited evidence (i.e., the CC of 2 μg/ml set by the World Health Organization [WHO] is higher than the ECOFF) (9, 29, 31).

Setting conclusive ECOFFs and validating MABA as a method for routine DST were beyond the scope of this study, which would have required a larger number of phylogenetically diverse strains from multiple laboratories and more extensive reproducibility testing, as specified by EUCAST and the International Organization for Standardization (ISO) (12, 28, 32, 33). Nevertheless, our MABA results were sufficiently robust compared with those of LJ DST and the genotypic results to set tentative ECOFFs. Accordingly, AFX had a lower ECOFF than OFX *in vitro* but an ECOFF higher than the ECOFFs of LFX and MFX. All *gyrA* mutations correlated with non-wild-type MICs for all fluoroquinolones. Consequently, clinicians should consider the possibility that the use of AFX to treat infections caused by E. coli, K. pneumoniae, and staphylococci at the doses currently suggested might result in the selection of fluoroquinolone resistance in M. tuberculosis in coinfected patients.

We had only one strain that had a *gyrB* mutation without a mutation in *gyrA*. The fact that four different amino acid changes had been observed at the *gyrB* codon in question (T500A/I/N/P) constitutes a potential signal for drug selection (7, 34, 35). In line with this observation, allelic exchange experiments with T500N in an Erdman background increased the MIC from wild-type levels to the CC for OFX and LFX and just above the CC for MFX (36). The results of the equivalent experiment in an H37Rv background were identical for OFX and LFX, but no increase in MIC was observed for MFX (36). In accordance with the results of the *in vitro* selection experiments and the aforementioned allelic exchange experiments, this suggested that the MIC of the strain with *gyrB* T500N was close to the ECOFF, which, due to biological and technical variability (e.g., for reproducibility, the ISO guidelines allow ±1 dilution of the mode for ≥95% of the results), would likely result in a poor reproducibility of DST (32, 37 – 39). Irrespective of whether this slightly elevated MIC increases the likelihood of treatment failure, it is possible that it increases the likelihood of selecting for higher levels of fluoroquinolone resistance due to a *gyrA* mutation or a secondary *gyrB* mutation, as observed for streptomycin (36, 40, 41). Larger data sets, ideally with longitudinal samples from the same patients, would be required to clarify this possibility (i.e., to determine in which order *gyrA* and *gyrB* mutations arose in double mutants, such as the five strains observed in this study [Fig. 1; see also Table S1 in the supplemental material]).

Using the published area under the concentration-time curve from time zero to 24 h (AUC_0–24_) of 47.59 ± 7.85 mg · h/liter for the currently approved dose of AFX (i.e., a 200-mg daily dose following a 400-mg loading dose) and protein binding of 17.5%, the unbound *f*AUC_0–24_/MIC ratio for the wild-type MICs of 0.064 to 1 μg/ml would range from 613.46 ± 101.19 h to 39.26 ± 6.48 h (42, 43). Although there is no consensus on the precise *f*AUC_0–24_/MIC ratio that best predicts *in vivo* efficacy, ratios of >100 at the upper end of the wild-type distribution are likely required to maximize clinical success (44, 45). Given that the currently recommended dose of AFX is unusually low (probably because of a narrow clinical indication) compared with the doses of the other fluoroquinolones used to treat tuberculosis, the target *f*AUC_0–24_/MIC of >100 at an increased dose is likely achievable, but this would have to be evaluated in clinical trials, where side effects would have to be monitored carefully.

Our study also has implications for DST for OFX on LJ. Although the absolute concentration method has not been validated by the WHO for second-line drugs, it is used clinically with the CC recommended for the proportion method (29). In our case, we employed a CC of 2 μg/ml, which corresponded to the old CC for this drug for the proportion method, which the WHO recently increased to 4 μg/ml, although the rationale for this change is unclear (29). In light of the excellent correlation between the LJ DST results and MABA MICs for all four fluoroquinolones, which is in line with the findings of previous studies, this suggested that the revised CC is likely too high for the absolute concentration method, resulting in non-wild-type strains being misclassified as wild type (46, 47). This, together with prior studies that raised doubts regarding the validity of some CCs, underlined the fact that the WHO should start to apply modern microbiological principles and, crucially, to publish the evidence used to set CCs, as has been the case for EUCAST for many years (12, 27, 39).

## Supplementary Material

Supplemental material

## References

[1] Wang J, Xiao Y, Huang W, Xu N, Bai C, Xiu Q, Mei C, Zheng Q 2010 A phase II study of antofloxacin hydrochloride, a novel fluoroquinolone, for the treatment of acute bacterial infections. Chemotherapy 56:378–385. doi:10.1159/000317581. 20938175

[2] Ge Y, Sun H, Wang M 2011 Crystal structure and fluorescence property of antofloxacin. J Southeast Univ 27:449–451.

[3] Franzblau SG, Witzig RS, McLaughlin JC, Torres P, Madico G, Hernandez A, Degnan MT, Cook MB, Quenzer VK, Ferguson RM, Gilman RH 1998 Rapid, low-technology MIC determination with clinical Mycobacterium tuberculosis isolates by using the microplate Alamar Blue assay. J Clin Microbiol 36:362–366. 9466742 10.1128/jcm.36.2.362-366.1998 PMC104543

[4] Yu X, Jiang G, Li H, Zhao Y, Zhang H, Zhao L, Ma Y, Coulter C, Huang H 2011 Rifampin stability in 7H9 broth and Löwenstein-Jensen medium. J Clin Microbiol 49:784–789. doi:10.1128/JCM.01951-10. 21177895 PMC3067696

[5] Dauendorffer JN, Guillemin I, Aubry A, Truffot-Pernot C, Sougakoff W, Jarlier V, Cambau E 2003 Identification of mycobacterial species by PCR sequencing of quinolone resistance-determining regions of DNA gyrase genes. J Clin Microbiol 41:1311–1315. doi:10.1128/JCM.41.3.1311-1315.2003. 12624075 PMC150312

[6] Shi R, Zhang J, Li C, Kazumi Y, Sugawara I 2006 Emergence of ofloxacin resistance in Mycobacterium tuberculosis clinical isolates from China as determined by *gyrA* mutation analysis using denaturing high-pressure liquid chromatography and DNA sequencing. J Clin Microbiol 44:4566–4568. doi:10.1128/JCM.01916-06. 17035499 PMC1698392

[7] Maruri F, Sterling TR, Kaiga AW, Blackman A, van der Heijden YF, Mayer C, Cambau E, Aubry A 2012 A systematic review of gyrase mutations associated with fluoroquinolone-resistant Mycobacterium tuberculosis and a proposed gyrase numbering system. J Antimicrob Chemother 67:819–831. doi:10.1093/jac/dkr566. 22279180 PMC3299416

[8] Bloemberg GV, Keller PM, Stuckia D, Trauner A, Borrell S, Latshang T, Coscolla M, Rothe T, Homke R, Ritter C, Feldmann J, Schulthess B, Gagneux S, Böttger EC 2015 Acquired resistance to bedaquiline and delamanid in therapy for tuberculosis. N Engl J Med 373:1986–1988. doi:10.1056/NEJMc1505196. 26559594 PMC4681277

[9] Willby M, Sikes RD, Malik S, Metchock B, Posey JE 2015 Correlation between *gyrA* substitutions and ofloxacin, levofloxacin, and moxifloxacin cross-resistance in Mycobacterium tuberculosis. Antimicrob Agents Chemother 59:5427–5434. doi:10.1128/AAC.00662-15. 26100699 PMC4538465

[10] Li XY, Li Y, Zhang Y, Kang WL, Zhao LP, Ding PJ, Dai WT, Huang HR, Huang YF, Li WM 2015 The epidemiological characteristics of Beijing lineage Mycobacterium tuberculosis from a national referral center in China. Biomed Environ Sci 28:539–543. doi:10.3967/bes2015.077. 26248739

[11] Comas I, Homolka S, Niemann S, Gagneux S 2009 Genotyping of genetically monomorphic bacteria: DNA sequencing in Mycobacterium tuberculosis highlights the limitations of current methodologies. PLoS One 4:e7815. doi:10.1371/journal.pone.0007815. 19915672 PMC2772813

[12] Kahlmeter G 2015 The 2014 Garrod Lecture: EUCAST—are we heading towards international agreement? J Antimicrob Chemother 70:2427–2439. doi:10.1093/jac/dkv145. 26089441

[13] Feuerriegel S, Köser CU, Niemann S 2014 Phylogenetic polymorphisms in antibiotic resistance genes of the Mycobacterium tuberculosis complex. J Antimicrob Chemother 69:1205–1210. doi:10.1093/jac/dkt535. 24458512

[14] Nebenzahl-Guimaraes H, Jacobson KR, Farhat MR, Murray MB 2014 Systematic review of allelic exchange experiments aimed at identifying mutations that confer drug resistance in Mycobacterium tuberculosis. J Antimicrob Chemother 69:331–342. doi:10.1093/jac/dkt358. 24055765 PMC3886931

[15] Avalos E, Catanzaro D, Catanzaro A, Ganiats T, Brodine S, Alcaraz J, Rodwell T 2015 Frequency and geographic distribution of *gyrA* and *gyrB* mutations associated with fluoroquinolone resistance in clinical Mycobacterium tuberculosis isolates: a systematic review. PLoS One 10:e0120470. doi:10.1371/journal.pone.0120470. 25816236 PMC4376704

[16] Hillemann D, Rüsch-Gerdes S, Richter E 2009 Feasibility of the GenoType MTBDR*sl* assay for fluoroquinolone, amikacin-capreomycin, and ethambutol resistance testing of Mycobacterium tuberculosis strains and clinical specimens. J Clin Microbiol 47:1767–1772. doi:10.1128/JCM.00081-09. 19386845 PMC2691112

[17] Mitarai S, Kato S, Ogata H, Aono A, Chikamatsu K, Mizuno K, Toyota E, Sejimo A, Suzuki K, Yoshida S, Saito T, Moriya A, Fujita A, Sato S, Matsumoto T, Ano H, Suetake T, Kondo Y, Kirikae T, Mori T 2012 Comprehensive multicenter evaluation of a new line probe assay kit for identification of Mycobacterium species and detection of drug-resistant Mycobacterium tuberculosis. J Clin Microbiol 50:884–890. doi:10.1128/JCM.05638-11. 22205814 PMC3295174

[18] Park C, Sung N, Hwang S, Jeon J, Won Y, Min J, Kim CT, Kang H 2012 Evaluation of reverse hybridization assay for detecting fluoroquinolone and kanamycin resistance in multidrug-resistance Mycobacterium tuberculosis clinical isolates. Tuberc Respir Dis 72:44–49. doi:10.4046/trd.2012.72.1.44.

[19] Ritter C, Lucke K, Sirgel FA, Warren RW, van Helden PD, Böttger EC, Bloemberg GV 2014 Evaluation of the AID TB resistance line probe assay for rapid detection of genetic alterations associated with drug resistance in Mycobacterium tuberculosis strains. J Clin Microbiol 52:940–946. doi:10.1128/JCM.02597-13. 24403306 PMC3957743

[20] Lee YS, Kang MR, Jung H, Choi SB, Jo KW, Shim TS 2015 Performance of REBA MTB-XDR to detect extensively drug-resistant tuberculosis in an intermediate-burden country. J Infect Chemother 21:346–351. doi:10.1016/j.jiac.2014.12.009. 25634305

[21] Molina-Moya B, Lacoma A, Prat C, Pimkina E, Diaz J, Garcia-Sierra N, Haba L, Maldonado J, Samper S, Ruiz-Manzano J, Ausina V, Dominguez J 2015 Diagnostic accuracy study of multiplex PCR for detecting tuberculosis drug resistance. J Infect 71:220–230. doi:10.1016/j.jinf.2015.03.011. 25936742

[22] Tagliani E, Cabibbe AM, Miotto P, Borroni E, Toro JC, Mansjo M, Hoffner S, Hillemann D, Zalutskaya A, Skrahina A, Cirillo DM 2015 Diagnostic performance of the new version of GenoType MTBDR*sl* (v2.0) assay for detection of resistance to fluoroquinolones and second line injectable drugs: a multicenter study. J Clin Microbiol 53:2961–2969. doi:10.1128/JCM.01257-15. 26179309 PMC4540937

[23] Canetti G, Froman S, Grosset J, Hauduroy P, Langerova M, Mahler HT, Meissner G, Mitchison DA, Sula L 1963 Mycobacteria: laboratory methods for testing drug sensitivity and resistance. Bull World Health Organ 29:565–578. 14102034 PMC2555065

[24] Folkvardsen DB, Svensson E, Thomsen VO, Rasmussen EM, Bang D, Werngren J, Hoffner S, Hillemann D, Rigouts L 2013 Can molecular methods detect 1% isoniazid resistance in Mycobacterium tuberculosis? J Clin Microbiol 51:1596–1599. doi:10.1128/JCM.00472-13. 23447641 PMC3647910

[25] Folkvardsen DB, Thomsen VO, Rigouts L, Rasmussen EM, Bang D, Bernaerts G, Werngren J, Toro JC, Hoffner S, Hillemann D, Svensson E 2013 Rifampin heteroresistance in Mycobacterium tuberculosis cultures as detected by phenotypic and genotypic drug susceptibility test methods. J Clin Microbiol 51:4220–4222. doi:10.1128/JCM.01602-13. 24068005 PMC3838044

[26] Pantel A, Petrella S, Veziris N, Brossier F, Bastian S, Jarlier V, Mayer C, Aubry A 2012 Extending the definition of the GyrB quinolone resistance-determining region in Mycobacterium tuberculosis DNA gyrase for assessing fluoroquinolone resistance in M. tuberculosis. Antimicrob Agents Chemother 56:1990–1996. doi:10.1128/AAC.06272-11. 22290942 PMC3318379

[27] Ängeby K, Juréen P, Kahlmeter G, Hoffner SE, Schön T 2012 Challenging a dogma: antimicrobial susceptibility testing breakpoints for Mycobacterium tuberculosis. Bull World Health Organ 90:693–698. doi:10.2471/BLT.11.096644. 22984314 PMC3442398

[28] Köser CU, Feuerriegel S, Summers DK, Archer JA, Niemann S 2012 Importance of the genetic diversity within the Mycobacterium tuberculosis complex for the development of novel antibiotics and diagnostic tests of drug resistance. Antimicrob Agents Chemother 56:6080–6087. doi:10.1128/AAC.01641-12. 23006760 PMC3497208

[29] World Health Organization. 2014 Companion handbook to the WHO guidelines for the programmatic management of drug-resistant tuberculosis. World Health Organization, Geneva, Switzerland http://apps.who.int/iris/bitstream/10665/130918/1/9789241548809_eng.pdf?ua=1&ua=1 Accessed 13 August 2015. 25320836

[30] Valsesia G, Roos M, Böttger EC, Hombach M 2015 A statistical approach for determination of disk diffusion-based cutoff values for systematic characterization of wild-type and non-wild-type bacterial populations in antimicrobial susceptibility testing. J Clin Microbiol 53:1812–1822. doi:10.1128/JCM.03506-14. 25762772 PMC4432064

[31] Rigouts L, Coeck N, Gumusboga M, de Rijk WB, Aung KJ, Hossain MA, Fissette K, Rieder HL, Meehan CJ, de Jong BC, Van Deun A 2016 Specific *gyrA* gene mutations predict poor treatment outcome in MDR-TB. J Antimicrob Chemother 71:314–323. doi:10.1093/jac/dkv360. 26604243 PMC4710215

[32] International Organization for Standardization. 2007 ISO 20776-2:2007. Clinical laboratory testing and *in vitro* diagnostic test systems—susceptibility testing of infectious agents and evaluation of performance of antimicrobial susceptibility test devices. Part 2. Evaluation of performance of antimicrobial susceptibility test devices, 1st ed International Organization for Standardization, Geneva, Switzerland.

[33] Torrea G, Coeck N, Desmaretz C, Van De Parre T, Van Poucke T, Lounis N, de Jong BC, Rigouts L 2015 Bedaquiline susceptibility testing of Mycobacterium tuberculosis in an automated liquid culture system. J Antimicrob Chemother 70:2300–2305. doi:10.1093/jac/dkv117. 25977401

[34] Farhat MR, Shapiro BJ, Kieser KJ, Sultana R, Jacobson KR, Victor TC, Warren RM, Streicher EM, Calver A, Sloutsky A, Kaur D, Posey JE, Plikaytis B, Oggioni MR, Gardy JL, Johnston JC, Rodrigues M, Tang PK, Kato-Maeda M, Borowsky ML, Muddukrishna B, Kreiswirth BN, Kurepina N, Galagan J, Gagneux S, Birren B, Rubin EJ, Lander ES, Sabeti PC, Murray M 2013 Genomic analysis identifies targets of convergent positive selection in drug-resistant Mycobacterium tuberculosis. Nat Genet 45:1183–1189. doi:10.1038/ng.2747. 23995135 PMC3887553

[35] Zhao LL, Sun Q, Zeng CY, Chen Y, Zhao B, Liu HC, Xia Q, Zhao XQ, Jiao WW, Li GL, Wan KL 2015 Molecular characterisation of extensively drug-resistant Mycobacterium tuberculosis isolates in China. Int J Antimicrob Agents 45:137–143. doi:10.1016/j.ijantimicag.2014.09.018. 25465521

[36] Malik S, Willby M, Sikes D, Tsodikov OV, Posey JE 2012 New insights into fluoroquinolone resistance in Mycobacterium tuberculosis: functional genetic analysis of *gyrA* and *gyrB* mutations. PLoS One 7:e39754. doi:10.1371/journal.pone.0039754. 22761889 PMC3386181

[37] McGrath M, Gey van Pittius NC, Sirgel FA, Van Helden PD, Warren RM 2014 Moxifloxacin retains antimycobacterial activity in the presence of *gyrA* mutations. Antimicrob Agents Chemother 58:2912–2915. doi:10.1128/AAC.02583-13. 24514091 PMC3993264

[38] Hombach M, Ochoa C, Maurer FP, Pfiffner T, Böttger EC, Furrer R 2016 Relative contribution of biological variation and technical variables to zone diameter variations of disc diffusion susceptibility testing. J Antimicrob Chemother 71:141–151. doi:10.1093/jac/dkv309. 26462987

[39] Niward K, Ängeby K, Chryssanthou E, Paues J, Bruchfeld J, Juréen P, Giske CG, Kahlmeter G, Schön T 2016 Susceptibility testing breakpoints for Mycobacterium tuberculosis categorize isolates with resistance mutations in *gyrA* as susceptible to fluoroquinolones: implications for MDR-TB treatment and the definition of XDR-TB. J Antimicrob Chemother 71:333–338. doi:10.1093/jac/dkv353. 26538509

[40] Okamoto S, Tamaru A, Nakajima C, Nishimura K, Tanaka Y, Tokuyama S, Suzuki Y, Ochi K 2007 Loss of a conserved 7-methylguanosine modification in 16S rRNA confers low-level streptomycin resistance in bacteria. Mol Microbiol 63:1096–1106. doi:10.1111/j.1365-2958.2006.05585.x. 17238915

[41] Reeves AZ, Campbell PJ, Sultana R, Malik S, Murray M, Plikaytis BB, Shinnick TM, Posey JE 2013 Aminoglycoside cross-resistance in Mycobacterium tuberculosis due to mutations in the 5′ untranslated region of *whiB7*. Antimicrob Agents Chemother 57:1857–1865. doi:10.1128/AAC.02191-12. 23380727 PMC3623337

[42] Liu L, Pan X, Liu HY, Liu XD, Yang HW, Xie L, Cheng JL, Fan HW, Xiao DW 2011 Modulation of pharmacokinetics of theophylline by antofloxacin, a novel 8-amino-fluoroquinolone, in humans. Acta Pharmacol Sin 32:1285–1293. doi:10.1038/aps.2011.78. 21892200 PMC4010077

[43] Lü Y, Kang ZS, Zhu Y, Zhang M, Liu Y, Zhang M, Li TY, Xiao YH 2011 Pharmacokinetic study of single and multiple oral dose administration of antofloxacin hydrochloride in healthy male volunteers. Chin Med J (Engl) 124:242–245. 21362374

[44] Ängeby KA, Juréen P, Giske CG, Chryssanthou E, Sturegård E, Nordvall M, Johansson AG, Werngren J, Kahlmeter G, Hoffner SE, Schön T 2010 Wild-type MIC distributions of four fluoroquinolones active against Mycobacterium tuberculosis in relation to current critical concentrations and available pharmacokinetic and pharmacodynamic data. J Antimicrob Chemother 65:946–952. doi:10.1093/jac/dkq091. 20332195

[45] Pranger AD, Alffenaar JW, Aarnoutse RE 2011 Fluoroquinolones, the cornerstone of treatment of drug-resistant tuberculosis: a pharmacokinetic and pharmacodynamic approach. Curr Pharm Des 17:2900–2930. doi:10.2174/138161211797470200. 21834759

[46] Nosova EY, Bukatina AA, Isaeva YD, Makarova MV, Galkina KY, Moroz AM 2013 Analysis of mutations in the *gyrA* and *gyrB* genes and their association with the resistance of Mycobacterium tuberculosis to levofloxacin, moxifloxacin and gatifloxacin. J Med Microbiol 62:108–113. doi:10.1099/jmm.0.046821-0. 23019190

[47] Singh P, Jain A, Dixit P, Prakash S, Jaiswal I, Venkatesh V, Singh M 2015 Prevalence of *gyrA* and *B* gene mutations in fluoroquinolone-resistant and -sensitive clinical isolates of Mycobacterium tuberculosis and their relationship with MIC of ofloxacin. J Antibiot (Tokyo) 68:63–66. doi:10.1038/ja.2014.95. 25052485

