# Supplementary material for: Wild-Type and Non-Wild-Type Mycobacterium tuberculosis MIC Distributions for the Novel Fluoroquinolone Antofloxacin Compared with Those for Ofloxacin, Levofloxacin, and Moxifloxacin
Source: Antimicrob Agents Chemother. 2016 Aug 22;60(9):5232–7. doi: 10.1128/AAC.00393-16 (PMC4997829; doi:10.1128/AAC.00393-16)
Supplement: Supplemental material [file supp_60_9_5232__index.html]

Wild-Type and Non-Wild-Type Mycobacterium tuberculosis MIC Distributions for the Novel Fluoroquinolone Antofloxacin Compared with Those for Ofloxacin, Levofloxacin, and Moxifloxacin — Supplemental material 

# Wild-Type and Non-Wild-Type Mycobacterium tuberculosis MIC Distributions for the Novel Fluoroquinolone Antofloxacin Compared with Those for Ofloxacin, Levofloxacin, and Moxifloxacin

## Supplemental material

- Supplemental file 1 -

  Table S1

  XLS, 70K
